# Association of the leptin-to-adiponectin ratio with metabolic syndrome in a sub-Saharan African population

**DOI:** 10.1186/s13098-017-0265-6

**Published:** 2017-09-05

**Authors:** Clarisse Noël A. Ayina, Francky Teddy A. Endomba, Samuel Honoré Mandengue, Jean Jacques N. Noubiap, Laurent Serge Etoundi Ngoa, Philippe Boudou, Jean-François Gautier, Jean Claude Mbanya, Eugene Sobngwi

**Affiliations:** 1 Department of Animal Science, Faculty of Science, University of Douala, Douala, Cameroon; 2 Department of Internal Medicine and Specialties, Faculty of Medicine and Biomedical Science, University of Yaoundé I, Yaoundé, Cameroon; 3 Department of Medicine, Groote Schuur Hospital, University of Cape Town, Cape Town, South Africa; 4 Department of Animal Science, Higher Teacher’s Training College, University of Yaoundé I, Yaounde, Cameroon; 5 Department of Hormonal Biology, Saint-Louis Hospital, Public Assistance -Paris Hospitals, University Paris-Diderot Paris-7, Paris, France; 6 Department of Diabetes and Endocrinology, Lariboisiere Hospital, Public Assistance – Paris Hospitals, University Paris-Diderot Paris-7, Paris, France; 7 INSERM UMRS 1138, Cordeliers Research Centre, University Pierre et Marie Curie-Paris 6, Paris, France; 8 Laboratory for Molecular Medicine and Metabolism, Biotechnology Center, University of Yaoundé I, Yaoundé, Cameroon; 9 National Obesity Center, Yaoundé Central Hospital, Yaoundé, Cameroon

**Keywords:** Adiponectin, Leptin, Leptin-to-adiponectin ratio, Metabolic syndrome

## Abstract

**Background:**

Worldwide there is an increased prevalence of metabolic syndrome mainly due to life-style modifications, and Africans are not saved of this situation. Many markers have been studied to predict the risk of this syndrome but the most used are leptin and adiponectin. Data on these metabolic markers are scare in Africa and this study aimed to assess the association between the leptin-to-adiponectin ratio (LAR) with metabolic syndrome in a Cameroonian population.

**Methods:**

This was a cross-sectional study that included 476 adults among a general population of Cameroon. Data collected concerned the body mass index, waist circumference, systolic blood pressure, diastolic blood pressure, fasting blood glucose, plasma lipids, adiponectin, leptin, insulin and homeostasis model for assessment of insulin resistance (HOMA-IR). To assess correlations we used Spearman’s analyses and association of the studied variables with metabolic syndrome were done using binary logistic regression analysis.

**Results:**

The leptin to adiponectin ratio was significantly and positively correlated with the body mass index (r = 0.669, p < 0.0001), waist circumference (r = 0.595, p < 0.0001), triglycerides (r = 0.190, p = 0.001), insulin levels (r = 0.333, p < 0.0001) and HOMA-IR (r = 0.306, p < 0.0001). Binary logistic regression analysis revealed that leptin, adiponectin and LAR were significantly associated with metabolic syndrome with respective unadjusted OR of 1.429, 0.468 and 1.502. After adjustment, for age and sex, the associations remained significative; LAR was also found to be significantly associated with metabolic syndrome (OR = 1.573, p value =0.000) as well as lower levels of adiponectin (OR = 0.359, p value =0.000) and higher levels of leptin (OR = 1.469, p value =0.001).

**Conclusion:**

This study revealed that LAR is significantly associated with metabolic syndrome in sub-Saharan African population, independently to age and sex.

## Background

There is a surge in the global prevalence of metabolic syndrome and its components including obesity, insulin resistance, diabetes mellitus, dyslipidemia and hypertension, as a result of reduced physical activity and unhealthy diet [1–4].The burden of metabolic syndrome has been rising over the past decade in African populations, driven by urbanization, sedentarity and nutritional transition [5–7]. To assess people at risk for the development of metabolic syndrome, several markers have been studied, with the large majority of them related to the adipose tissue [3–6]. Two markers are of particular interest, adiponectin and leptin, which are strongly associated to cardiometabolic health or disease [1, 3, 7, 8]. For instance, in obese patients who are at risk of insulin resistance and thus metabolic syndrome, secretion of adiponectin by hypertrophic adipocytes is reduced, while that of leptin is increased [12–14]. Indeed, leptin and adiponectin levels have been found to be respectively positively and negatively correlated with obesity, diabetes mellitus, hypertension and metabolic syndrome [12–15].Leptin and adiponectin also have opposite’s effects on inflammatory markers and thus subclinical inflammation [9]. Leptin is considered as a proinflammatory cytokine since it up regulates pro-inflammatory cytokines such as TNF-α and IL-6 [9–11]. On the contrary, adiponectin displays anti-inflammatory properties by down regulation of the expression and release of proinflammatory mediators [9, 12–14].

Recent studies have demonstrated in the potential of the leptin-to-adiponectin ratio (LAR) as a novel predictor of cardio-metabolic outcomes including metabolic syndrome [3, 15–17]. LAR has been shown to be associated with insulin resistance, metabolic syndrome, carotid intima-media thickness, “at-risk phenotype” in young severely obese patients, and chronic kidney disease, among others [18–20]. Some others studies found this marker to be a better tool for the diagnosis of metabolic syndrome and risk stratification of subjects, than adiponectin or leptin alone [15, 16, 21]. In a recent study we demonstrated that leptin and adiponectin correlate to surrogates of obesity, blood lipids and insulin resistance in sub-Saharan Africans [22]. The current study aimed investigates the association of LAR with metabolic syndrome in a group of Cameroonians.

## Methods

### Study population

This is a cross-sectional study conducted in May 2010 in Douala and Edéa, two urban cities of the Littoral region of Cameroon. The study population consisted of individuals of both sex aged ≥18 years from the general population, who accepted to participate in the study after an invitation through radio announcements. Pregnant and breast-feeding women, as well as subjects with serious chronic illness, or ongoing or recent (<10 days) acute illness, or those taking any current medication were excluded from the study. After applying the exclusion criteria, 476 participants were included in the study.

### Data collection

For each subject, weight was measured in light clothes with a seca scale balance to the nearest 0.1 kg, height with a calibrated stadiometer, waist circumference (WC) at midway between the lowest rib and the iliac crest and hip circumference at the outermost points of the greater trochanters to the nearest 0.5 cm and waist-to-hip ratio (WHR) as waist circumference divided by hip circumference. Body mass index (BMI) was calculated using the Quetelet’s formula as weight (in kg) divided by height (in m^2^). We measured resting blood pressures twice using standardized procedures with the participant in a seated position, and after at least 10 min rest with a validated automated blood pressure measuring device, the Omron HEM-757 (Omron Corporation, Tokyo, Japan). The mean of two measures performed at least 3 min apart was used for all analyses. Percentage body fat (%BF) was measured by bioelectric impedance analysis using the OMRON BF 302 (OMRON Matsusaka Co., Tokyo, Japan). After 8–12 h overnight fast, blood glucose was measured between 7 and 10 a.m. using the Accu-Chek^®^ compact plus glucometer (F. Hoffmann-La Roche AG, Basel, Switzerland) on total fresh capillary blood samples, and venous blood samples were obtained from an antecubital vein. Serum was then separated and stored at −20 °C for lipid measurements and at −80 °C for further biochemical analysis. Serum lipids were analyzed within 1 week after the collection. Serum cholesterol (TC) (cholesterol oxidase phenol 4-amino antipyrine peroxidase method), serum triglycerides (TG) (glycerol phosphatase oxidase–phenol4-amino antipyrine peroxidase method), and high-density lipoprotein-cholesterol (HDL-c) (cholesterol oxidase phenol4-amino antipyrine peroxidase method) were measured on a spectrophotometer (UV mini 1240) using Chronolab kits (Chronolab Systems, Barcelona, Spain). Low-density lipoprotein-cholesterol (LDL-c) was calculated using the Friedwald’s formula. Insulin levels were measured using an electrochemiluminescence immunoassay (Roche Diagnostics, Indianapolis, USA), while serum leptin and adiponectin were measured by radio immuno assay (RIA) using Linco Research kits (Linco Research Inc., St Charles, Missouri, USA) with the following characteristics: Adiponectin assay coefficient of variation (CV) ≤10%; detection limit of 2 ng/mL for 100 µl sample size; a linearity range of 500 ng/mL for 100 µL sample size. Leptin assay’s coefficient of variation ≤10% (intra-assay’s CV ≤8.3% and inter assay’s CV ≤6.2%); a detection limit of 0.5 ng/mL for 100 µL sample size; a linearity range of 100 ng/mL for 100 µL sample size. Anthropometrical and biochemical measurements was made once on each participant.

Homeostasis model assessment of insulin resistance (HOMA-IR) as

$${\text{HOMA}} - {\text{IR}} = \left( {{\text{Fasting insulin}}\left( {\mu \frac{\text{UI}}{\text{mL}}} \right) \times {\text{Fasting blood glucose}}\left( {\frac{\text{Mmol}}{\text{L}}} \right)} \right) \div 22.5$$

### Definition of anthropomorphic indices of obesity

According to the World Health Organization guidelines, obesity was defined as BMI ≥30 kg/m^2^ [23], WC >94 cm in men and >80 cm in women, WHR ≥0.90 in men and ≥0.85 in women [24]. The BF% cutoffs chosen to define obesity were the values most frequently cited by international scientific literature: BF%  ≥25 for men and ≥35 for women [25].

### Definition of metabolic syndrome

The metabolic syndrome was defined using, the IDF/AHA/NHLBI consensus harmonized definition published by Alberti et al. in 2009 [26], that recommends three or more of any of the following criteria: WC ≥80 cm (women) ≥94 cm (men), SBP ≥130 mmHg and/or DBP ≥85 mmHg, or blood pressure lowering treatment, fasting plasma glucose ≥5.6 mmol/L [101 mg/dL], or anti diabetic treatment, fasting triglycerides >1.70 mmol/L [154.5 mg/dL] or triglyceride lowering drugs and HDL-c <1 mmol/L [40 mg/dL] (women) <1.3 mmol/L [52 mg/dL] (men).

### Statistical analysis

Data were coded, entered and analyzed using the statistical package for social science (SPSS) version 20.0 for Windows (IBM Corp. Released 2011. IBM SPSS statistics for windows, version 20.0. Armonk, NY: IBM Corp.). The distribution pattern of the variables was checked. Normally distributed variables are expressed as mean with standard deviation (SD). Skewed variables are reported as median (interquartile range). Skewed variables were log transformed. The L/A ratios are expressed as the absolute value of plasma leptin (ng/mL) divided by the absolute value of plasma adiponectin (μg/mL). The independent sample t test was used to compare clinical and biological parameters between men and women. Spearman’s correlations were used to determine correlates of leptin and adiponectin serum levels, as well as LAR’s. Binary logistic regression analysis included adiponectin, leptin and LAR as dependent variables, and were used to identify independent factors able to predict metabolic syndrome after adjustment for age, sex and BMI. Statistical tests are two tailed. A *p* value <0.05 was considered statistically significant.

### Ethical considerations

This study was performed in accordance with the guidelines of the Helsinki declaration and was approved by the national ethics committee for human health research and by the ministry of public health of Cameroon. Written informed consent was obtained from all participants.

## Results

### Clinical and metabolic characteristics of the study population

A total of 476 subjects (167 men and 309 women) were enrolled in the study. The median age (interquartile) was 55.0 (23.6) for men and 50.0 (20.0) for women (*p* < 0.05). WHR, SBP and DBP were significantly higher in men (*p* < 0.05 respectively). Triglycerides, LDL-c, insulin, adiponectin, leptin, and HOMA-IR were significantly higher in women (*p* < 0.05 respectively) (Table 1).

**Table 1:** Clinical and metabolic characteristics of the study population)

	Men (n = 167)	Women (n = 309)	*p* value
Age	55 ± 23	50 ± 20	<0.0001
Weight (kg)	71.00 ± 23.00	70.00 ± 25.00	0.984
Height (m)	1.70 ± 0.20	1.60 ± 0.10	<0.0001
BMI (kg/m^2^)	25.30 ± 6.30	28.00 ± 6.60	<0.0001
WC (cm)	91.04 ± 12.6	93.58 ± 14.0	0.091
WHR (cm)	0.91 ± 0.09	0.87 ± 0.09	<0.0001
BF (%)	22.90 ± 8.62	34.00 ± 10.42	<0.0001
SBP (mm Hg)	137 ± 37	*128* ± *33*	<0.0001
DBP (mm Hg)	86 ± 21	82 ± 20	0.006
Blood glucose (mmol/L) [mg/dL]	5.0 ± 1.10 [90.9 ± 20]	4.80 ± 1.15 [87.3 ± 20.9]	0.204
Total cholesterol (mmol/L) [mg/dL]	4.14 ± 1.07 [165.6 ± 42.8]	4.45 ± 1.16 [178 ± 46.4]	0.05
Triglycerides (mmol/L) [mg/dL]	1.20 ± 0.45 [109.9 ± 40.9]	1.26 ± 0.35 [114.5 ± 31.8]	0.024
LDL-c (mmol/L) [mg/dL]	1.48 ± 0.97 [59.2 ± 38.8]	1.78 ± 1.17 [71.2 ± 46.8]	0.031
HDL-c (mmol/L) [mg/dL]	2.13 ± 0.99 [85.2 ± 39.6]	2.14 ± 0.91 [85.6 ± 36.4]	0.763
Insulin (µU/mL)	2.14 ± 2.16	2.73 ± 2.47	0.020
HOMA-IR	8.64 ± 0.25	11.05 ± 11.13	0.031
Adiponectin (µg/mL)	7.65 ± 5.55	10.75 ± 8.90	0.038
Leptin (ng/mL)	4.80 ± 6.20	18.50 ± 24.40	<0.0001

WC values are means (standard deviation); except for WC, values are median (interquartile range)

*WC* waist circumference, *BF%* body fat percentage, *BMI* body mass index, *WHR* waist-hip ratio, *SBP* systolic blood pressure, *DBP* diastolic blood pressure, *LDL*-*c* low density lipoprotein-cholesterol, *HDL*-*c* high density lipoprotein-cholesterol, *HOMA*-*IR* homeostasis model assessment of insulin resistance

### Correlations between leptin-to-adiponectin ratio and anthropometric indexes of obesity, blood pressure, blood lipids and insulin resistance

As represented in Table 2, leptin-to-adiponectin ratio was significantly and positively correlated with body mass index (r = 0.669, p < 0.0001), waist circumference (r = 0.595, p < 0.0001), triglycerides (r = 0.190, p = 0.001), insulin levels (r = 0.333, p < 0.0001) and HOMA-IR (r = 0.306, p < 0.0001). There was no significant correlation within LAR and systolic or diastolic blood pressure, glycaemia and HDL cholesterol.

**Table 2:** Pearson’s correlation of adiponectin, leptin and LAR with parameters of metabolic syndrome

	Adiponectin		Leptin		LAR	
	R	*p*	R	*P*	r	*p*
IMC (kg/m^2^)	−0.297*	0.000	0.649*	0.000	0.669*	0.000
WC (cm)	−0.297*	0.000	0.560*	0.000	0.595*	0.000
SBP (mm Hg)	0.013	0.820	0.045	0.416	0.034	0. 537
DBP (mm Hg)	−0.005	0.921	0.116*	0.036	0.099	0.073
Blood glucose (mmol/L)	−0.042	0.443	−0.014	0.797	0.013	0.814
TG (mmol/L)	−0.140*	0.011	0.150*	0.007	0.190*	0.001
HDL-c (mmol/L)	0.095	0.087	−0.012	0.833	−0.058	0.296
Insulin (µU/mL)	−0.123	0.059	0.332*	0.000	0.333*	0.000
HOMA-IR	−0.139*	0.032	0.288*	0.000	0.306*	0.000

*WC* waist circumference, *BMI* body mass index, *SBP* systolic blood pressure, *DBP* diastolic blood pressure, *TG* triglycerides, *HDL*-*c* high density lipoprotein-cholesterol, *HOMA*-*IR* homeostasis model assessment of insulin resistance, * Significant correlation

### Association between leptin-to-adiponectin ratio and metabolic syndrome

Binary logistic regression analysis (see Table 3) metabolic syndrome was significantly associated with higher levels of leptin (OR = 1.42, p value =0.003) as well as with lower levels of adiponectin (OR = 0.46, p value =0.001) and LAR (OR = 1.5, p value <0.0001). After adjustment for age and sex, the LAR was also found to be significantly associated with metabolic syndrome (OR = 1.573, p value =0.000) as well as lower levels of adiponectin (OR = 0.359, p value =0.000) and higher levels of leptin (OR = 1.469, p value =0.001).

**Table 3:** Unadjusted and adjusted (for age and sex) ORs (95% CI) for metabolic syndrome according to log-transformed leptin, adiponectin and LAR, by binary logistic regression analysis

Variables	Metabolic syndrome			
	Unadjusted OR (CI)	*p*	Adjusted OR (CI)	*p*
Leptin	1.429 (1.134–1.802)	0.003	1.469 (1.16–1.86)	0.001
Adiponectin	0.468 (0.304–0.720)	0.001	0.359 (0.225–0.573)	0.000
LAR	1.502 (1.231–1.833)	0.000	1.573 (1.281–1.932)	0.000

## Discussion

It’s nowadays accepted that adipokines mostly leptin and adiponectin, play an important role in many physiological pathways including glucose metabolism through insulin regulation [4, 27]. It has been demonstrated that these molecules can be predictive of metabolic syndrome. Indeed lower adiponectin levels have been found to be significantly associated with metabolic syndrome [1, 3, 7, 8]. In the other hand, metabolic syndrome is found to be associated with higher levels of leptin. Even though these markers are well described in literature, some recent findings revealed the possible role of leptin-to-adiponectin ratio in the prediction of cardiometabolic diseases including metabolic syndrome [3, 15–17]. This study was conducted to so assess the relationship of LAR with some anthropometric indexes, blood lipids, blood pressure and insulin resistance among a sub-Saharan African population.

Our study revealed a positive correlation of LAR to body mass index, waist circumference, triglycerides and insulin resistance. These findings are similar to those of Kotani et al. [28] in a study that involved 678 non-smoking Japanese subjects. The authors found that the LAR was significantly and positively associated with components of metabolic syndrome including BMI and triglycerides, especially in men. Nevertheless, contrary to them, no significant correlation was found between glucose levels and LAR. Zyl et al. [29] found similar results in their study on South African urban women, although LAR was significantly higher in women with elevated blood glucose. This finding seems to be specific to Africans. In the literature, it has been proved that LAR is associated with increased waist circumference and also decreased vascular response to acetylcholine [9, 15]. Also, the LAR has been found to be associated with an increased vasoconstrictive response to angiotensin II [30].

With and without adjustment for age and sex, metabolic syndrome was significantly associated with LAR, leptin and adiponectin. Thus the LAR levels were significantly higher in metabolic syndrome subjects than in non metabolic syndrome, as well as the levels of leptin. Meanwhile, adiponectin levels were significantly lower in subjects with metabolic syndrome. These results look like those of Kotani et al. [28] who found that LAR was significantly higher in peoples with metabolic syndrome, even when adjusted for sex. Zhuo et al. [16] in a study that involved 950 males and 1096 females aged between 60 and 96 years; found that LAR, as well as leptin, may be better markers than adiponectin for the diagnosis of metabolic syndrome. Also, the authors found that LAR has better capacity in the classification of subjects with and without metabolic syndrome than adiponectin or leptin alone. Many others studies have noticed the function of LAR as a good marker of obesity, diabetes mellitus, insulin resistance and metabolic syndrome compared to adiponectin or leptin alone [6, 19, 21]. LAR has also been found to be related to low grade inflammation and insulin resistance independently of obesity, with a more powerful association with CRP and HOMA-IR than leptin or adiponectin alone [6, 31]. Our study has a principal strength that this population was not receiving potentially confounding medications during the investigation. Nevertheless, this study has two main limitations. First, its cross-sectional design prevented us from identifying cause-and-effect associations LAR and MS. Secondly, we used HOMA-IR to evaluate insulin sensitivity rather than gold-standard methods such as the hyperinsulinemic euglycemic clamp and the frequently sampled intravenous glucose tolerance test which, however, would have been significantly very difficult to perform in such a population-based study in a resource-limited setting. Despite these limitations, our study appears to be the first of this type among a sub-Saharan African population, and noted the possible role of LAR as a diagnostic marker of MS in black Africans peoples.

## Conclusion

In this study higher levels of LAR have been found to be significantly associated with metabolic syndrome as well as higher levels of leptin and lowered ones of adiponectin. Thus, studies with appropriate design must be carried out to really assess whether LAR can be a predictive factor of metabolic syndrome or not, independently of confounding factors.

## Abbreviations

BF%: percent body fat
BMI: body mass index
CRP: C-reactive protein
HDL-c: high-density lipoprotein cholesterol
HOMA-IR: homeostasis model for assessment of insulin resistance
IL: interleukin
LAR: leptin-to-adiponectin ratio
LDL-c: low-density lipoprotein cholesterol
TC: total cholesterol
TG: triglycerides
TNF-α: tumor necrosis factor alpha
WC: waist circumference
WHR: waist-to-hip ratio
